# The Corrosion Characteristics and Tensile Behavior of Reinforcement under Coupled Carbonation and Static Loading

**DOI:** 10.3390/ma8125479

**Published:** 2015-12-09

**Authors:** Yidong Xu

**Affiliations:** School of Civil Engineering & Architecture, Ningbo Institute of Technology of Zhejiang University, Ningbo 315100, China; xyd@nit.zju.edu.cn; Tel.: +86-138-5748-8759; Fax: +86-574-8822-9132

**Keywords:** reinforcement, carbonation, static loading, coupled effect, non-uniform corrosion, corrosion morphology, finite element analysis

## Abstract

This paper describes the non-uniform corrosion characteristics and mechanical properties of reinforcement under coupled action of carbonation and static loading. The two parameters, namely area-box (AB) value and arithmetical mean deviation (*R*_a_), are adopted to characterize the corrosion morphology and pitting distribution from experimental observations. The results show that the static loading affects the corrosion characteristics of reinforcement. Local stress concentration in corroded reinforcement caused by tensile stress drives the corrosion pit pattern to be more irregular. The orthogonal test results from finite element simulations show that pit shape and pit depth are the two significant factors affecting the tensile behavior of reinforcement. Under the condition of similar corrosion mass loss ratio, the maximum plastic strain of corroded reinforcement increases with the increase of *R*_a_ and load time-history significantly.

## 1. Introduction

The corrosion of reinforcement is one of the major deterioration mechanisms for reinforced concrete (RC) structures. Once reinforcement corrodes, its mechanical properties are affected and as a consequence the durability and serviceability of RC structures. In order to promote the effective application of reinforced concrete and to ensure that an RC structure has good performance during its service life, it becomes necessary to understand the mechanisms of how reinforcement corrodes in a given environment and how its deterioration affects the performance of the structure.

Considerable research work has been carried out on the description of morphology and distribution of corroded metal. Rivas [1] applied extreme value analysis to pitting corrosion experiments in low-carbon steel. Caleyo and Valor [2,3,4] described the evolution of pitting depth by using Weibull, Fréchet, and Gumbel distribution functions. A Monte Carlo simulation was proposed by Caleyo [2] to estimate the probability distributions of the maximum pit depth and pit growth rate. Codaro [5] and Demis [6] developed a digital image processing and analysis method to evaluate the shape and size of corrosion pits. Horner [7] and Huang [8] adopted X-ray microtomography and confocal imaging profiler to observe the three-dimensional corrosion pit morphology of 3NiCrMoV disc steel and 7075-T6 aluminum alloy. Cerit [9] investigated the stress concentration factor (SCF) at the semielliptical corrosion pit by using finite element analysis. Pidaparti [10] analyzed the morphological features of corroded aluminum 5059 alloy samples by using a statistical method, and finite element analysis was adopted to predict surface stresses distribution.

Many researchers have investigated the bond behavior [11,12,13] and concrete cover cracking due to corrosion expansion [14,15,16,17,18]. The work of Apostolopoulos is quite extensive in this field [19,20,21,22,23,24]. By using accelerated electrochemical corrosion tests, Almusallam [25] found that reinforcing bars with 12.6% or more reinforcement corrosion have brittle behavior. Du [26] discussed the influence of type, diameter and corrosion time of reinforcing bars on their residual capacity. Moreno [27] established the mathematical models to predict the mechanical behavior of reinforcements depending on the corrosion degree and diameter of the rebars. Cobo [28] conducted the experiment on the mechanical properties of corroded B500SD high ductility reinforcement. Results show that the elongation of the bars diminishes and the ratio between the maximum tensile stress and the elastic limit increases as the corrosion degree advances. Zhang [29] conducted an experimental study on the static tensile and fatigue behavior of corroded reinforcement. Compared with the tensile behavior, the fatigue behavior of corroded reinforcement was more affected by corrosion. Souza [30] established a damage evolution model of corroded reinforcement by using damage mechanicals. 

The above-mentioned studies, however, are mainly focused on the effects of chemical corrosion, such as chloride and carbonation, yet the coupled chemo-mechanical process has not been considered. Furthermore, the influence of pitting geometry and distribution on the tensile behavior of corroded reinforcement was not thoroughly discussed apart from a few exemptions [6].

The aim of this paper is to investigate the relationship between the macro- and meso-material characteristics, in which the former is related to the mechanical weakening of the material and the latter is associated with a large number of randomly distributed corrosion pits of irregular shapes, sizes, and orientations. In particular, it studies whether there is any difference for the corrosion characteristics of the reinforcement under carbonation attack and under the coupled effect of carbonation and static loading, and what sort of impact the different corrosion characteristics have on the tensile behavior of the reinforcement. Based on stereological method and surface roughness characterization technology, in this work the pitting geometry and distribution of reinforcement under coupled effects of carbonation and static loading have been characterized. The effect of different pitting geometry and distribution on the tensile behavior of reinforcement has been analyzed using finite element analysis.

## 2. Corrosion Characteristics of Reinforcement under Different Corrosion Conditions

### 2.1. Materials

The designed concrete compressive strength (measured on cubes) is 30 MPa, and the actual measurement is 36.2 MPa, according to Chinese Standards GB/T 50081 [31]. The mix proportion of concrete is shown in Table 1. The cement used was Type 42.5 Portland cement according to Chinese Standards GB175 [32]. The fine aggregate used was river sand. The coarse aggregate used was natural dolomite gravel with a maximum diameter of 31.5 mm. Hot rolled plain steel bar of type HPB235 (with nominal diameter of 12 mm) was used as reinforcement. When the corrosion test was completed, the reinforcements were taken out from the concrete and washed using a rust removing solution to remove corrosion products. The rust removing solution was prepared by mixing 3 parts by mass of hexamethylene tetramine into 97 parts diluted hydrochloric acid. 

**Table 1:** Mix proportion of concrete specimen (kg/m^3^).

Portland Cement	Water	Fine Aggregate	Coarse Aggregate
365	192	730	1095

### 2.2. Accelerated Corrosion Test Procedures

**Figure 1:**
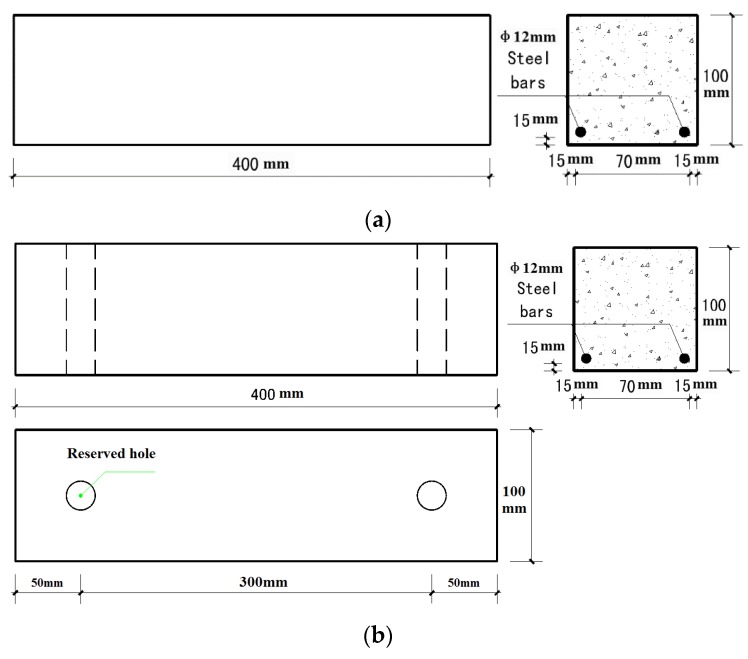
Details of the tested reinforced concrete specimens: (**a**) RT (the specimen under carbonation attack); (**b**) RTL (the specimen under the coupled action of carbonation and static loading).

Specimens under carbonation attack (denoted as RT) and under the coupled action of carbonation and static loading (denoted as RTL) were prepared. Figure 1 shows the details of the reinforced concrete specimens tested. After curing in a fog room (20 ± 2 °C, 95% relative humidity) for 28 days, the specimens were demolded and then used for the accelerated corrosion test. 

**Figure 2:**
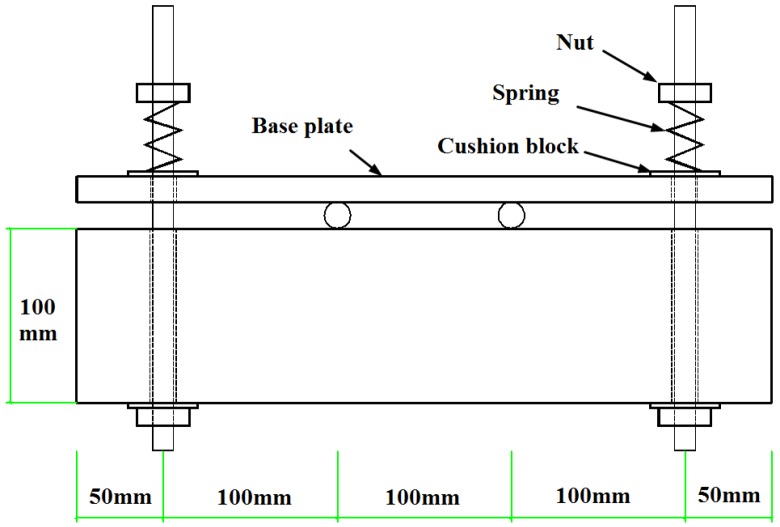
Schematic illustrations of the loading frame.

The RTL specimen was loaded by using a self-equilibrium loading frame, shown in Figure 2. In the loading system, the tested concrete specimen was connected to a steel plate (base plate) using two spring-controlled loading bolts, and was thus subjected to a four-point bending. The magnitude of the load applied was 1.6 kN (90% of the cracking load), which was calculated following the method described in ref. [33], in order to prevent cracking in concrete. The applied load was measured by the deformation of a spring. In order to prevent the stress relaxation of the spring, load correction was conducted every week in the first month, and then the correction procedure was conducted every month until all the specimens were tested.

Four RT specimens and another four RTL specimens were prepared for this experiment. All of the tested specimens were placed into a carbonation tank with 20% ± 3% CO_2_ concentration and 70% ± 5% RH to cause the corrosion of reinforcement in concrete. According to the experimental plan, each pair of RT and RTL specimens was broken every three months. After the carbonation tests, the reinforcing bars were removed from the concrete and washed by using the rust removing solution to remove corrosion products, from which the corrosion mass loss ratio of the reinforcement (denoted as *S*) was calculated. The detailed corrosion mass loss ratios are shown in Table 2.

**Table 2:** Corrosion mass loss ratio (*S*) of corroded reinforcement.

Code	*S/*%	Code	*S/*%
RT-P1	1.10	RTL-P1	0.69
RT-P2	1.99	RTL-P2	1.55
RT-P3	2.10	RTL-P3	2.51
RT-P4	3.65	RTL-P4	3.73

### 2.3. Image Acquisition and Characterization of Corrosion Morphology

The corrosion morphology images of the reinforcement were taken by using a ME-61 stereomicroscope (Micro-shot Technology Co., Guangzhou, China) with magnification of 7×, which covered the central region of 100 mm long in the corroded part [34]. 

**Figure 3:**
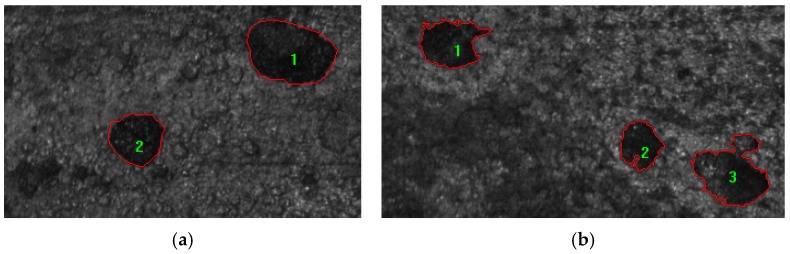
Typical pit patterns for different AB parameter: (**a**) Circular pits, AB parameter for the two pits is 0.73; (**b**) Transition zone A pits, AB parameters are 0.63, 0.65 and 0.59 for the three pits, respectively.

To simplify the description of pitting geometry, a classical shape descriptor parameter, namely, the area-box (AB) parameter was used [34,35]. The AB parameter is defined as the ratio between the pit area and the area of minor surrounding rectangle that encloses the pit, which facilitates the characterization of pitting morphology, as is shown in Table 3. The typical pit patterns are shown in Figure 3. For calculating the AB parameter, the Image Pro Plus 6.0 (Media Cybernetics Inc., Rockville, MD, USA) was used.

**Table 3:** The corresponding area-box (AB) parameter for pit pattern with different geometrical characteristics.

AB Parameter Interval	0–0.5	0.5–0.54	0.54–0.72	0.72–0.86	0.86–0.98	0.98–1.0
corrosion pit pattern	irregular	triangles	transition zone A	circles	transition zone B	rectangles
geometrical characteristic	-	conical pits	-	spherical pits	-	cylindrical pits

### 2.4. Pitting Depth Acquisition and Characterization of Corroded Surface Profiles

Along the 100 mm length of the corroded reinforcement, pitting depths were measured using a surface profilometer (with accuracy of 0.01 mm, Sanhe Measuring Implement Co., Wenzhou, China) at intervals of 2.5 mm in the longitudinal direction and 45° in the radial direction. Eight curves along the axial direction, equally distributed on the reinforcement surface, were established.

**Figure 4:**
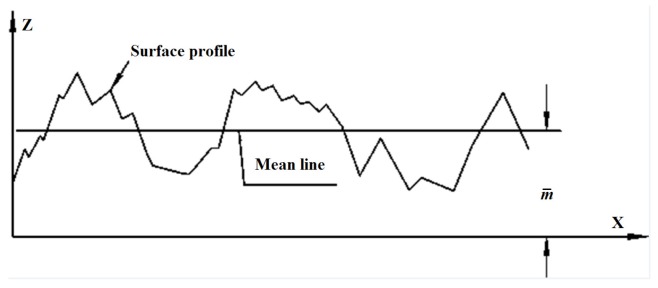
Schematic diagram of the arithmetical mean deviation (*R*_a_).

Researchers have proposed many statistical roughness parameters relating to the vertical features of a surface roughness profile, with the most widely used being the arithmetical mean deviation (denoted as *R*_a_) [36]. It is defined as the average absolute deviation of the roughness irregularities from mean line over one sampling length as shown in Figure 4. The mathematical definition and the digital implementation of *R*_a_ are shown in Equation (1) [37].

(1) $$R_{\text{a}}=\frac{1}{l}\int_{0}^{l}\left|z-\bar{m}\right|dx=\frac{1}{n}\sum_{i=1}^{n}\left|z-\bar{m}\right|$$

where *z* is the height of profile peak, $\bar{m}$ is the height of mean line, *l* is the sampling length, and *n* is the number of measuring points.

### 2.5. Results and Discussion

**Figure 5:**
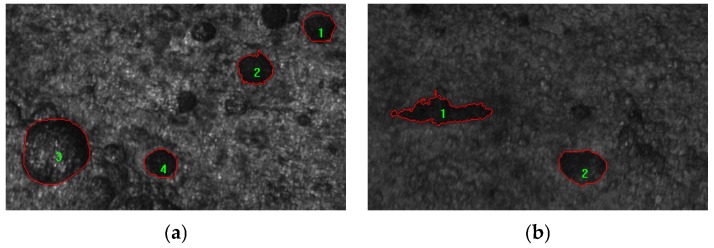
Typical pit patterns for reinforcement under different corrosion conditions: (**a**) RT specimen, AB parameters are 0.74, 0.68, 0.80 and 0.76 for the four pits, respectively; (**b**) RTL specimen, AB parameters are 0.44 and 0.74 for the two pits, respectively.

**Figure 6:**
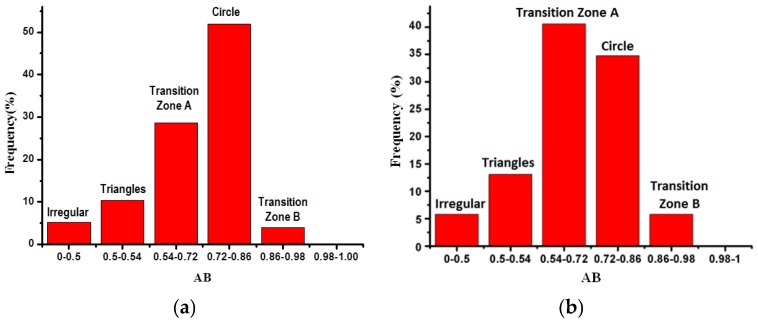
The proportion of AB parameters for different corroded reinforcement: (**a**) RT-P4; (**b**) RTL-P4.

The proportions of AB parameters for specimens under different corrosion conditions were analyzed by using frequency statistics; the changing tendency of corrosion characteristics of RT and RTL is similar. The typical pit patterns for RT and RTL specimens are shown in Figure 5. Here the experimental results of two specimens, RT-P4 and RTL-P4, are discussed because the two specimens have almost the same corrosion mass loss ratio. As is shown in Figure 6, the AB parameters for different specimens are mainly located in the following two intervals, [0.54, 0.72] and [0.72, 0.86]. Under carbonation attack, the proportion of circular shape pits in the reinforcement is the highest and the proportion of transition-zone-A shape pits is lower. Irregular, transition-zone-B, and triangle-shape pits take only very small proportions. While under the coupled action of carbonation and static loading, the proportion of transition-zone-A shape pits is slightly higher than that of the circle shape pits. The statistical results indicate that the tensile stress can make the corrosion pit pattern more complicated. The coupled action of carbonation and static loading affects the distribution of corrosion pit pattern. Indeed, previous works have also shown that pitting susceptibility and pitting potential in stainless steel is dependent on the type of load and the level of applied stress [38,39]. To evaluate if concrete carbonation can promote brittle fracture of pre-stressed reinforcement, constant load tests in bicarbonate aqueous solutions under anodic polarization were carried out by Proverbio [40], and deep internal voids close to the surface were observed. Owing to the local stress concentration in corroded reinforcement caused by tensile stress, the corrosion pits grow along the preferred orientation, which makes the corrosion pit pattern more irregular.

The maximum and average values of arithmetical mean deviation (*R*_a_) of surface profiles at eight lines as described above were calculated and are shown in Table 4. Under the condition of similar corrosion mass loss ratio (*S*), the surface roughness of the RT specimen is found to be lower than that of the RTL specimen, which indicates that the coupled chemo-mechanical process affects the distribution of pitting depth [41].

**Table 4:** Surface roughness of corroded reinforcement. *R*_a_: Arithmetical mean deviation.

Code	*S/*%	(*R*_a_)_ave_/μm	(*R*_a_)_max_/μm
RT-P4	3.65	55.21	92.00
RTL-P4	3.73	64.5	114.7

## 3. Influence of Pitting Geometry on the Tensile Behavior of Reinforcement

For reinforced concrete structures exposed to the atmospheric environment, corrosion relates mainly to the carbonation of concrete cover. Once CO_2_ penetrates in the concrete cover and reaches to the surface of reinforcement, the protective film around the reinforcement is broken and the active corrosion of steel occurs [42]. As is shown in Section 2.5, there exist a lot of pits in the reinforcement, and the pit pattern has different geometrical characteristics. The influence of pitting geometry on the tensile behavior of reinforcement has become a new focus. In this section, orthogonal array method and finite element analysis (FEA) are used to investigate this problem. 

### 3.1. Orthogonal Array of Pitting Geometry

Using orthogonal array testing, we can maximize the test coverage while minimizing the number of test cases to consider [43,44]. The experimental arrangement can be optimized by adopting appropriate arrays for different variables and levels. By using range analysis and variance analysis, the influence of every variable on experimental results can be described quantitatively. In this study, the orthogonal array L_9_(3^4^) was used for experimental arrangement and data analysis. The experimental runs with four parameters are operated using Orthogonality Experiment Assistant, a statistical software which incorporates a L_9_(3^4^) orthogonal array table. The software determines nine significant experimental runs instead of 81 runs in the case of 4 variables, with 3 levels each (3^4^ = 81) [45]. The selected physical variables are the pit shape, corrosion mass loss ratio, and pit depth. In order to estimate the "accidental error" occurring in the experiment, the variable of last column is usually adopted as blank, denoted as "error". The experimental levels are shown in Table 5.

The ductile fracture process of corroded reinforcement is as follows. Local plastic deformation develops along the pit, which causes a stress redistribution. The re-distributed stresses will affect the overall stress in the specimen, which can generate plastic deformation in other places. This could cause a necking and/or fracture with dimple cleavage in the weak place of the specimen. Obviously, the fracture of corroded reinforcement originates the excessive plastic deformation in pitting. Therefore, the maximum equivalent plastic strain (denoted as PEEQ in ABAQUS) is adopted as the index for this numerical simulation.

**Table 5:** Variables and levels for orthogonal test.

Level	Variables			
	(A) Pit Shape	(B) Corrosion Mass Loss Ratio(*S*)/%	(C) Pit Depth/mm	(D) Error
(a)	Spherical	1.0	1.0	-
(b)	Ellipsoidal	1.5	1.5	-
(c)	Triangular pyramid	2.0	2.0	-

### 3.2. Finite Element Model

**Figure 7:**
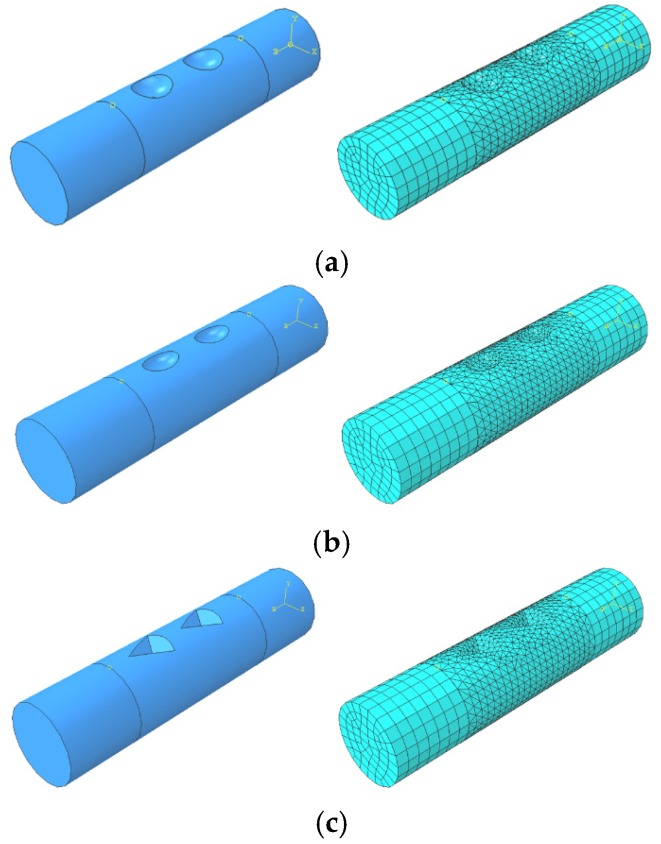
Finite element models: (**a**) FEA model of corroded reinforcement with circular pits; (**b**) FEA model of corroded reinforcement with ellipsoidal pits; (**c**) FEA model of corroded reinforcement with triangular pyramid pits.

**Figure 8:**
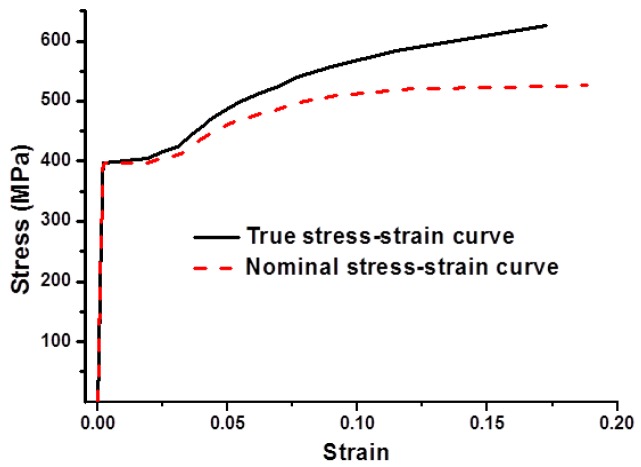
Stress-strain curves of uncorroded reinforcing bar.

Finite element analysis (FEA) was carried out using ABAQUS/Standard implicit solver and the models used are shown in Figure 7. The overall length of the specimen is 50 mm. The pit was assumed as spherical pit, ellipsoidal pit, and triangular pyramid pit, the size of which was determined based on three parameters: the size of the surface, the depth of the pit, and the volume of the pit. Owing to the existence of pits, the model was partitioned into three parts in order to generate different mesh grid. The two end regions of the model were meshed by using an eight-node brick element with reduced integration (C3D8R). The middle part of the model with pits was meshed by using a 10-node quadratic tetrahedral element (C3D10). A fine mesh was used in the middle part of the model in order to achieve accurate results. The boundary conditions employed are as follows: one end of the cylinder was assumed to be fully fixed and the other end was subjected to a tensile force. The applied displacement was 8.5 mm. The material used is classical metal plasticity and the related stress-strain data used are shown in Figure 8. The number of element used in each model was 9831, 13201, 12831, 18361, 22437, 16500, 29765 and 27830, respectively. These were based on the trials to achieve good accuracy but without increasing extra computational efforts.

When using ABAQUS for elastic-plastic analysis, the modeling of materials nonlinearity requires the definition of a true stress-strain relationship for isotropic metals [46]. Therefore, the nominal stress-strain relation should be converted to the true stress-strain relation. The transformation formula is shown in Equation (2):

(2) $$\varepsilon_{\text{true}}=\ln\left(1{+\varepsilon }_{\text{nom}}\right),{\;\sigma }_{\text{true}}{=\sigma }_{\text{nom}}\left(1{+\varepsilon }_{\text{nom}}\right)$$

where ε_true_ is the true strain, σ_true_ is the true stress, ε_nom_ is the nominal strain, and σ_nom_ is the nominal stress.

Tensile testing was conducted for the uncorroded reinforcement by using a servo-hydraulic testing machine. The detailed testing procedure is reported in a previous study [47]. The nominal and true stress-strain curves of the uncorroded reinforcement are shown in Figure 8, from which Young’s modulus and Poisson’s ratio were found to be *E*_s_ = 200 GPa and μ = 0.3.

### 3.3. Results and Discussion

**Figure 9:**
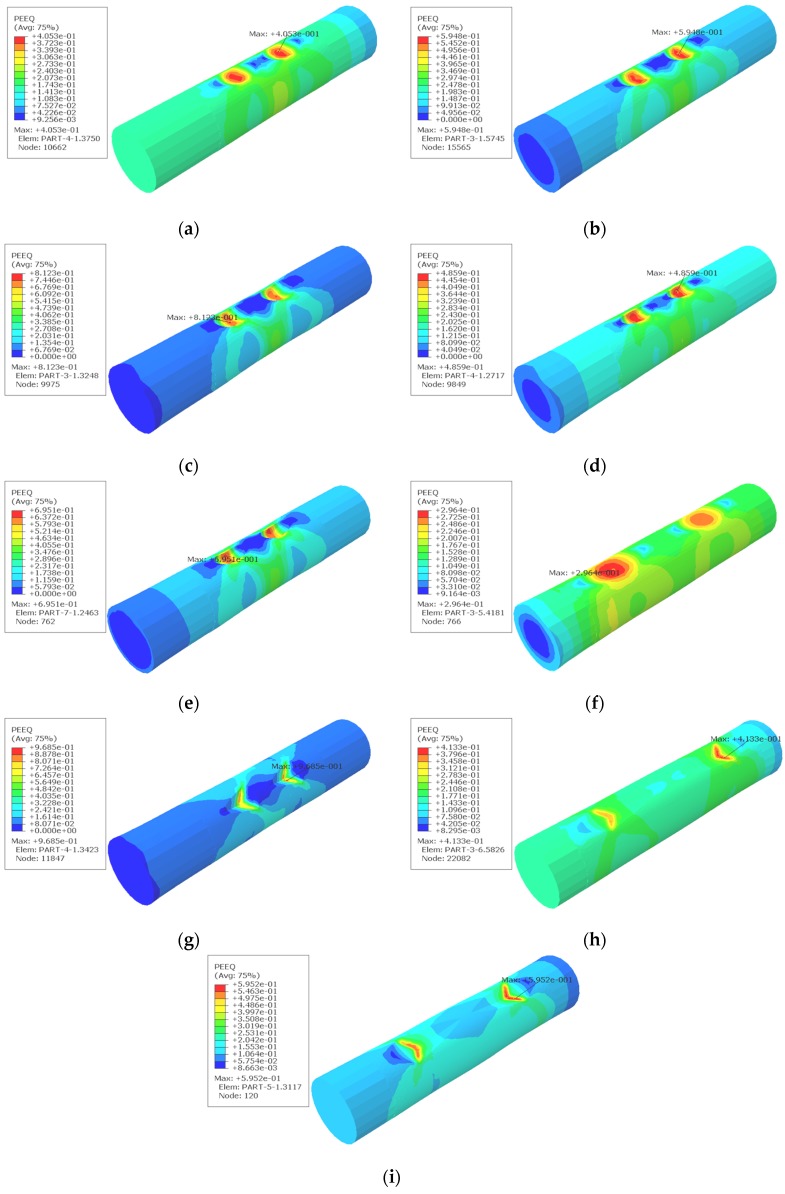
Results of equivalent plastic strains (PEEQ) obtained from FEA based on Table 6: (**a**) 1#; (**b**) 2#; (**c**) 3#; (**d**) 4#; (**e**) 5#; (**f**) 6#; (**g**) 7#; (**h**) 8#; (**i**) 9#.

The detailed conditions for FEA are shown in Table 6. As is shown in Figure 9, the existence of pits leads to stress concentration in the reinforcement, which causes to the deterioration of the plastic deformation of the reinforcement. The stress concentration around corrosion pits is obvious. As is expected, all of the maximum equivalent plastic strains (PEEQ) occur around the corrosion pits.

Table 6 and Table 7 show the range analysis and variance analysis of the maximum equivalent plastic strain (PEEQ). In the range analysis of orthogonal test, *K_i_* is the average PEEQ for a certain variable at level *i*, and by comparing to *K_i_*, the optimal level of variables can be confirmed. The definition of parameter *Y* is $\max\{K_{I},K_{II},K_{III}\}-\max\{K_{I},K_{II},K_{III}\}$, and *Y* scales the effect of variables on the result. A high *Y* value of a certain variable means that this variable has a relatively strong effect on the result [48,49]. Obviously, the *Y* value of column D (error) should be the minimum among the four columns, which means the experimental results are reliable. In the variance analysis, the *F*-test is sensitive to non-normality and the related *F* value is a key parameter in the *F*-test statistic. If the obtained F value is equal to or larger than the critical F value, then the result is significant at that level of probability. In this case, *F_0_*_.05_ = 6.94. Since *F*_Pit shape_ and *F*_Pit depth_ are all greater than 6.94, the results are significant at the 5% significance level. It is observed from the tables that the orders of influence of each variable are pit depth > pit shape > corrosion mass loss ratio > error. The two variables, pit depth and pit shape, are significant factors affecting the tensile behavior of reinforcement. The maximum equivalent plastic strain (PEEQ) increases remarkably with the increase of pit depth, which leads to the failure of corroded reinforcement under low loads. Since the *F* value of corrosion mass loss ratio is less than the critical value (*F*_0.05_), corrosion mass loss ratio is not a significant variable regarding the mechanical properties of reinforcement. Given the same amount of mass loss in reinforcement, localized corrosion appears to be the more dangerous damage type than uniform corrosion, owing to the localized and concentrated material deterioration, which causes local stress concentration and could lead to catastrophic failure of the material in that area.

**Table 6:** Experimental arrangement and range analysis. PEEQ: Equivalent plastic strain.

Experiment No.	(A)	(B)	(C)	(D)	Index
	Pit Shape	*S/*%	Pit Depth/mm	Error	PEEQ
1#	Spherical (a)	1.0 (a)	1.0 (a)	(a)	0.4053
2#	Spherical (a)	1.5 (b)	1.5 (b)	(b)	0.5948
3#	Spherical (a)	2.0 (c)	2.0 (c)	(c)	0.8123
4#	Ellipsoidal (b)	1.0 (a)	1.5 (b)	(c)	0.4859
5#	Ellipsoidal (b)	1.5 (b)	2.0 (c)	(a)	0.6951
6#	Ellipsoidal (b)	2.0 (c)	1.0 (a)	(b)	0.2964
7#	Triangular pyramid (c)	1.0 (a)	2.0 (c)	(b)	0.9687
8#	Triangular pyramid (c)	1.5 (b)	1.0 (a)	(c)	0.4133
9#	Triangular pyramid (c)	2.0 (c)	1.5 (b)	(a)	0.5952
**Range Analysis**	**PEEQ**	**PEEQ**	**PEEQ**	**PEEQ**	**-**
*K*_I_	0.609	0.624	0.376	0.570	
*K*_II_	0.492	0.568	0.559	0.620	
*K*_III_	0.659	0.568	0.825	0.571	
*Y*	0.167	0.056	0.449	0.05	

**Table 7:** Analysis of variance.

Source	Index	Sum of Squares of Deviations	Degree of Freedom	*F* Value	Critical Value
pit shape	PEEQ	0.044	2	8.00 ^**^	*F*_0.05_ = 6.94
corrosion mass loss ratio (*S*)	PEEQ	0.006	2	1.091	
pit depth	PEEQ	0.306	2	55.636 ^**^	

** means significantly affected.

**Figure 10:**
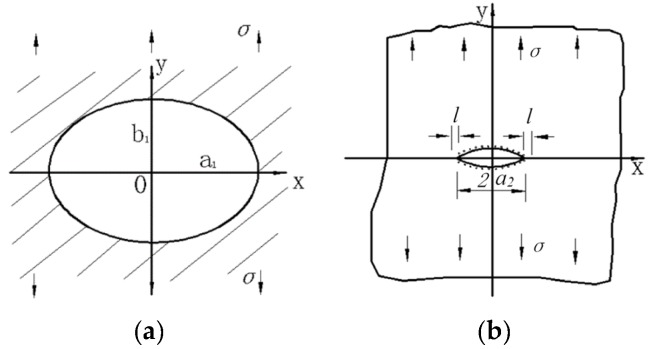
Elliptical hole defect of infinite-plate model: (**a**) Elliptical hole in infinite plate; (**b**) Crack propagation.

Different pit shape also has an obvious influence on the mechanical properties of the corroded reinforcement. A growing corrosion pit will essentially be inducing a corresponding increase in stress and strain [7]. Huang summarizes the stress concentration factor for different corrosion pit shape ratios. The stress concentration factor increases with the depth of the pit, and the stress tends to increase when the pit is more elongated in the direction normal to the applied load [8]. Figure 10 shows an ellipsoidal pit in a 2-dimensional infinite solid under far-field tensile forces [50]. According to the results of Inglis on the stress concentration around an elliptical hole [51], the stress distribution is described in Equation (3) and Equation (4). Based on three-dimensional reconstruction techniques, Azevedo [52] calculated the stress concentration factor of corroded SAE 2205 duplex stainless steel samples with 3D pits. The results showed that 3D reconstruction data of pits is valid for the calculation of stress concentration factors using the Inglis equation. In this sense, the use of the data of 3D pits along with FEA might lead to interesting calculations of stress concentration factor in irregular pits.

when λ ≠ 1,

(3) $$\sigma_{\text{b}}=\frac{\sigma }{1-\lambda }(\sqrt{{\left(1+\rho \right)}^{2}-\rho^{2}{\left(1-\lambda \right)}^{2}}-\lambda \rho )(\frac{1-2\lambda }{1-\lambda }+\frac{\lambda \rho }{\sqrt{{\left(1+\rho \right)}^{2}-\rho^{2}(1-\lambda^{2})}})+\frac{\lambda^{\text{2}}\sigma }{\left(1-\lambda^{2}\right)}$$

when λ = 1,

(4) $$\sigma_{\text{b}}=\frac{\sigma }{\text{2}}(2+\frac{\left(4\rho^{3}+5\rho^{2}+2\rho \right)}{{\left(1+\rho \right)}^{3}})$$

where σ_b_ is the largest stress concentration, σ is the macroscopic stress, λ = *b*_1_/*a*_1_, and ρ = *a*_1_/*l*, *a* and *b* are the major and minor axes, respectively.

**Figure 11:**
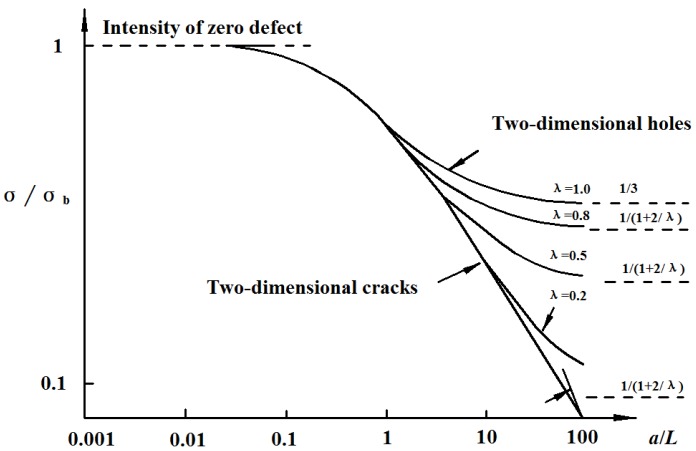
Influence of defect on failure stress.

Figure 11 shows the relationship between defect size and failure strength [50]. When λ = 1, an ellipsoidal hole reduces to a spherical hole and the strength of materials reaches its maximum. With the decrease of λ, an ellipsoidal hole gradually transits to a slender hole and the strength of materials decreases rapidly. It explains that a corrosion pit with a circle pattern has a blunt feature, which leads to less stress concentration. Obviously, a triangular pyramid-shaped pit is more irregular, which causes localized and concentrated material deterioration and leads to larger equivalent plastic strain.

To further discuss the effect of non-uniform corrosion on the mechanical properties of reinforcement, another FEA analysis was conducted. It is hard to establish different FEA models with identical corrosion mass loss ratios but with different pit distributions. Here, three models with different arithmetical mean deviations but with similar corrosion mass losses were constructed. The maximum difference of corrosion mass loss ratio is only 0.38%, as is shown in Table 8.

**Table 8:** Constructor parameters of corroded reinforcement.

Code	Pit Shape	*S/*%	*R*_a_ of Quadrant Surface-1/μm	*R*_a_ of Quadrant Surface-2/μm	*R*_a_ of Quadrant Surface-3/μm	*R*_a_ of Quadrant Surface-4/μm	(*R*_a_)_ave_/μm	(*R*_a_)_max_/μm
*R*_a_-1	Spherical	1.72	417	202	179	251	262	417
*R*_a_-2	Spherical	1.82	287	491	223	456	364	491
*R*_a_-3	Spherical	2.10	467	610	440	340	464	610

**Figure 12:**
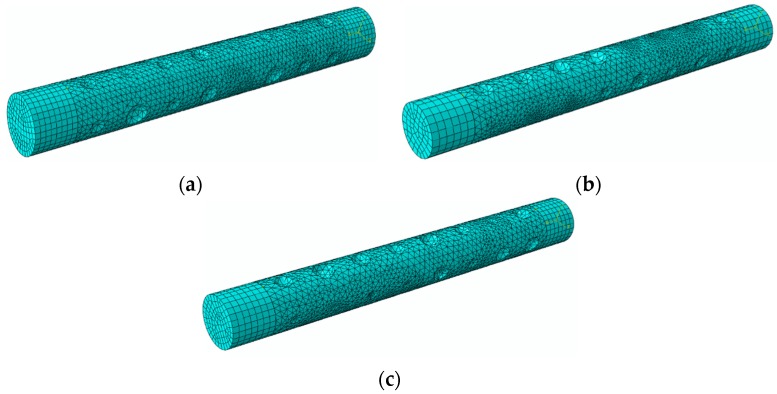
FEA models with different pit distributions based on Table 8: (**a**) *R*_a_-1; (**b**) *R*_a_-2; (**c**) *R*_a_-3.

**Figure 13:**
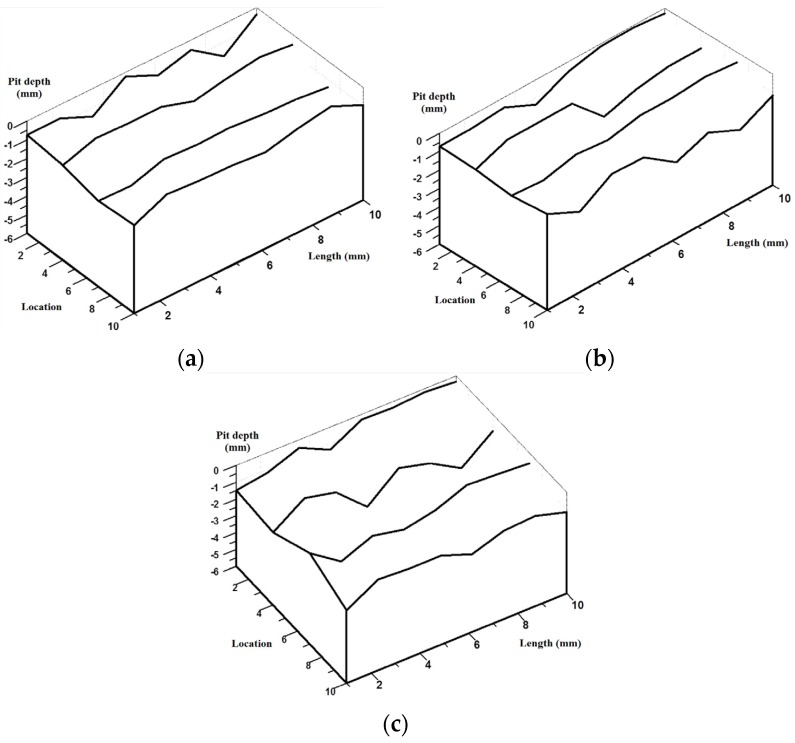
Surface profile curves of finite element models based on Table 8: (**a**) *R*_a_-1; (**b**) *R*_a_-2; (**c**) *R*_a_-3.

Figure 12 shows the finite element model with different pit distribution. The element type, mesh, boundary condition and constitution model are the same as those described in Section 3.2. The overall length of the model is 100 mm. The spherical pits are distributed on the surface profiles at four lines along the axial direction, equally distributed on the reinforcement surface. Each surface has eight pits with different depth. By using Equation (1), the related arithmetical mean deviation (*R*_a_) was calculated, as is shown in Table 8. Figure 13 plots the related surface profile curves of the three FEA models. It can be seen from the figure that, although the corrosion mass loss ratios (*S*) have no significant differences between the three models, the tortuosity of each curve is found to increase with increased arithmetical mean deviation (*R*_a_). This indicates that the non-uniform distribution of pit depth increases with surface roughness of reinforcement.

**Figure 14:**
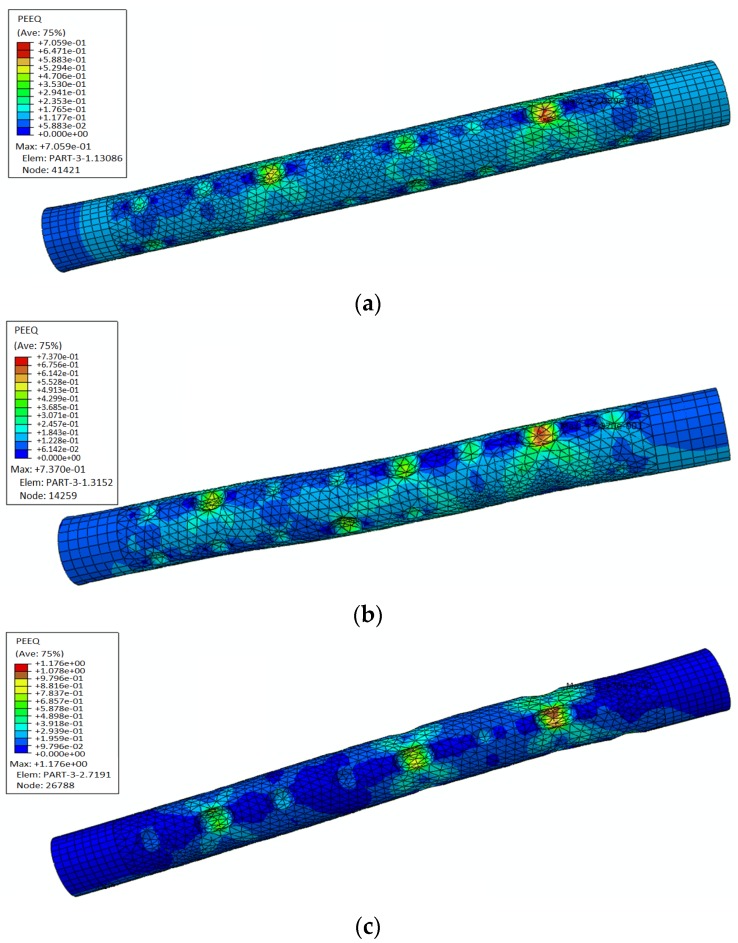
Results of finite element analysis based on Table 8: (**a**) *R*_a_-1; (**b**) *R*_a_-2; (**c**) *R*_a_-3.

**Figure 15:**
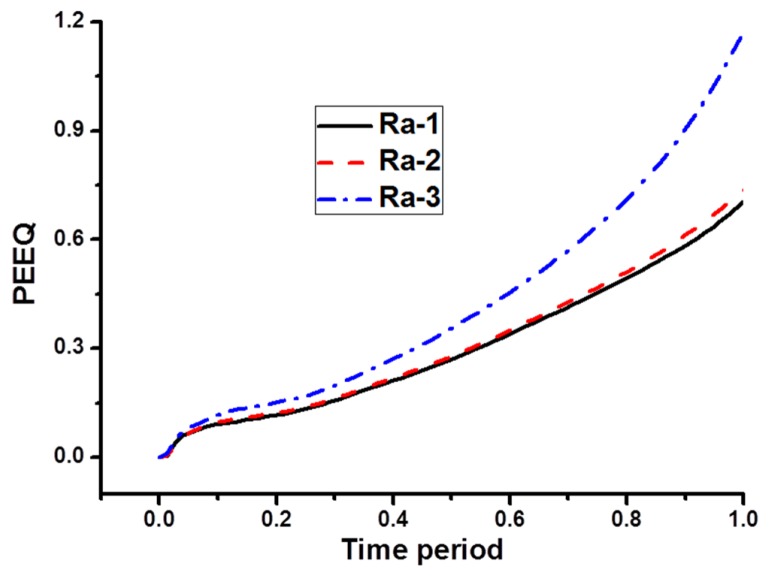
Load time-history of maximum equivalent plastic strain.

As is shown in Figure 14 and Figure 15, the non-uniform distribution of pits has a significant influence on the mechanical properties of the corroded reinforcement. In general, under the condition of similar corrosion mass loss ratios, the maximum equivalent plastic strain (PEEQ) of corroded reinforcement increases with the increase of *R*_a_. The maximum equivalent plastic strain (PEEQ) on *R*_a_-1 is 0.7059, hence 0.7059 increases up to 0.737 on *R_a_*-2. The increment is 4.4%. However, on *R*_a_-3 the PEEQ is 1.176; hence, compared to *R*_a_-1 is an increase of 66.5%, which is very substantial. As shown in Table 8, the maximum arithmetical mean deviation (*R*_a_)_max_ on *R*_a_-1 is 417 μm, hence 417 μm increases up to 491 μm on *R*_a_-2. The increment is 74 μm. However, on *R*_a_-3 the (*R*_a_)_max_ is 610; hence, compared to *R*_a_-2, an increase of 119 μm. Therefore, the influence of maximum arithmetical mean deviation (*R*_a_)_max_ may be the main reason for the rapid increment of PEEQ. The most disadvantageous location coincides with the pit with the maximum depth. The maximum equivalent plastic strain (PEEQ) increases generally with the increase of load time-history, which leads to the failure of corroded reinforcement under low loads.

**Figure 16:**
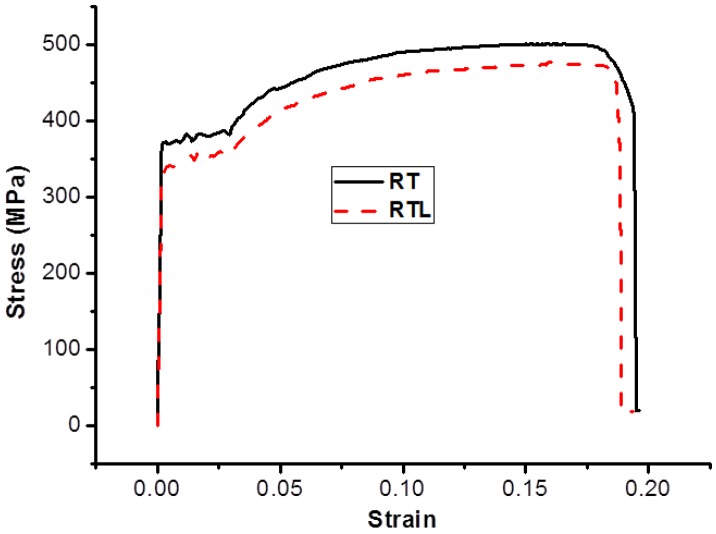
Stress-strain curves of RT and RTL specimens.

In order to verify the results of FEA, tensile tests of the two specimens, RT and RTL, was conducted. Figure 16 shows the stress-strain curves of the two reinforcements. It is observed from the figure that there exists an obvious difference in the strength. Owing to the more complicated corrosion pitting morphology of reinforcement under the combined effects of carbonation and static loading, the mechanical properties of RTL are remarkably lower than those of the RT specimen. Since the two specimens have similar corrosion mass loss ratios, the results indicate that the yield strength and ultimate strength of RTL reached 340 and 470 MPa, at values of 92% and 94% of RT, respectively.

## 4. Conclusions

This paper has presented an investigation of the corrosion characteristics and tensile behavior of reinforcement under the coupled action of carbonation and static loading. The experimental results showed that the existence of a static load affects the corrosion characteristics of the reinforcement significantly. Owing to the coupled action of carbonation and static loading, the corrosion pits grow along the preferred orientation, which makes the corrosion pit pattern more angular, and the non-uniform distribution of pit depth in the reinforcement increases significantly.

A finite element analysis performed showed that the existence of corrosion pits leads to stress concentration in the corroded reinforcement. The order of the influence of each variable are pit depth > pit shape > corrosion mass loss ratio > error. The two variables, pit depth and pit shape, are the significant factors affecting the maximum equivalent plastic strain. Under the condition of similar corrosion mass loss ratios, the maximum equivalent plastic strain of corroded reinforcement increases with the increase of *R*_a_ and load time-history. The tensile test has demonstrated that the yield strength and ultimate strength of RTL-P4 reached 340 and 470 MPa, at values of 92% and 94% of RT-P4, respectively.
